# Interleukins: Pathogenesis in Non-Alcoholic Fatty Liver Disease

**DOI:** 10.3390/metabo14030153

**Published:** 2024-03-06

**Authors:** Saira Rafaqat, Sanja Gluscevic, Filiz Mercantepe, Sana Rafaqat, Aleksandra Klisic

**Affiliations:** 1 Department of Zoology, Lahore College for Women University, Lahore 54600, Pakistan; 2 Clinical Center of Montenegro, Department for Neurology, 81000 Podgorica, Montenegro; 3 Department of Endocrinology and Metabolism, Faculty of Medicine, Recep Tayyip Erdogan University, 53010 Rize, Turkey; 4 Department of Biotechnology (Human Genetics), Lahore College for Women University, Lahore 54600, Pakistan; 5 Faculty of Medicine, University of Montenegro, 81000 Podgorica, Montenegro; 6 Center for Laboratory Diagnostics, Primary Health Care Center, 81000 Podgorica, Montenegro

**Keywords:** non-alcoholic fatty liver disease, interleukins, inflammation

## Abstract

Inflammatory cytokines have been implicated as crucial contributors to the onset and progression of non-alcoholic fatty liver disease (NAFLD). The exact mechanisms by which interleukins (ILs) contribute to NAFLD may vary, and ongoing research is aimed at understanding the specific roles of different ILs in the pathogenesis of this condition. In addition, variations in environmental factors and genetics in each individual can influence the onset and/or progression of NAFLD. The lack of clinical studies related to the potential therapeutic properties of IL-1 inhibitors currently does not allow us to conclude their validity as a therapeutic option, although preclinical studies show promising results. Further studies are needed to elucidate their beneficial properties in NAFLD treatment.

## 1. Introduction

Non-alcoholic fatty liver disease (NAFLD) stands as a predominant contributor to liver-related ailments on a global scale. The estimated occurrence of NAFLD worldwide is 47 instances per 1000 individuals, with a higher frequency observed in males than females. Among adults globally, the estimated prevalence of NAFLD is 32%, with a notable gender difference—40% in males compared to 26% in females. Over time, there has been a discernible rise in the global prevalence of NAFLD, ascending from 26% in research conducted before 2005 to 38% in research conducted from 2016 onward. This escalation in prevalence is marked by regional disparities, influenced by varying rates of obesity, as well as socioeconomic and genetic factors. Particularly, the prevalence of NAFLD surpasses 40% in south-east Asia and America [1].

Recently, the term “metabolic-associated fatty liver disease” (MAFLD) was used to rename non-alcoholic fatty liver disease (NAFLD), which is a hepatic manifestation of metabolic syndrome [2,3,4]. Insulin resistance and the lack of other causes of liver steatosis are what define it, with fat build-up occurring in over 5% of hepatocytes [2,5]. Between simple steatosis or non-alcoholic fatty liver (NAFL) and non-alcoholic steatohepatitis (NASH), NAFLD is a collection of histological abnormalities of variable severity and prognosis [2]. The pathophysiology of NAFLD is intricate and diverse, as evidenced by the clinical spectrum it encompasses, from simple steatosis to cirrhosis, representing the end stage of liver disease. Numerous factors contribute to the initiation of metabolic alterations in the liver. An excessive intake of nutrients can disrupt the balance of microorganisms in the gastrointestinal tract, leading to dysbiosis. This imbalance, coupled with increased permeability of the intestinal barrier, facilitates the translocation of microbial-associated molecular patterns to the liver through the portal vein. Consequently, these patterns enter the systemic circulation, triggering pro-inflammatory reactions within the liver. Additionally, specific dietary components can instigate mechanisms of the disease in liver tissue [3,4,5].

Numerous liver cell types, such as macrophages, neutrophils, diverse immune cells, and hepatocytes, actively participate in the inflammatory processes of fatty liver diseases. Additionally, microRNAs (miRNAs), extracellular vesicles (EVs), and the complement system contribute to the inflammatory milieu. The inflammatory response is further influenced by the liver’s inter-tissue interaction with other organs such as the gut, adipose tissue, and the neurological system. Inflammation also assumes crucial roles in facilitating liver repair and managing bacterial infections. Comprehending the intricate regulatory mechanisms that disrupt homeostasis during the progression of NAFLD holds the potential to unveil targeted therapeutic interventions. This understanding may pave the way for improved strategies aimed at intervening in the inflammatory processes associated with NAFLD, ultimately contributing to more effective and precise therapeutic approaches [6].

Interleukins (ILs) represent a class of cytokines initially believed to be exclusively expressed by leukocytes. However, subsequent discoveries have revealed their production by various other cells throughout the body. These molecules play crucial roles in the activation and differentiation of immune cells, as well as in processes such as proliferation, maturation, migration, and adhesion. Notably, ILs exhibit both pro-inflammatory and anti-inflammatory properties. The fundamental role of ILs lies in modulating growth, differentiation, and activation during inflammatory and immune responses. Comprising a diverse group of proteins, ILs can evoke a range of cellular and tissue responses by binding to high-affinity receptors on cell surfaces. Their functionality extends to both paracrine and autocrine mechanisms. In addition, ILs find utility in animal studies for exploring aspects related to clinical medicine [7].

**Figure 1:**
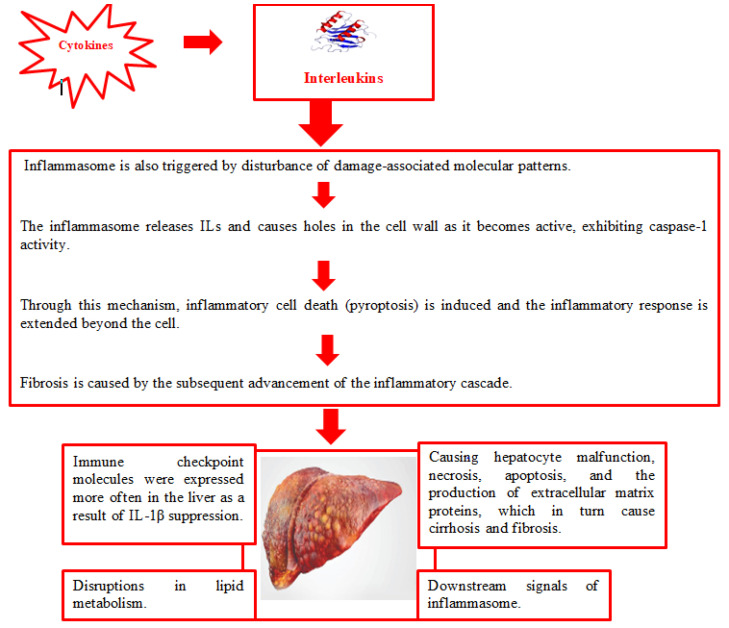
The role of interleukins in NAFLD.

ILs have been classified according to how they affect the inflammatory response in the scientific literature. Three separate categories result from this categorization, i.e., inflammatory cytokines, anti-inflammatory molecules, and interleukins having a dual function, that may operate as both inflammatory and anti-inflammatory molecules under certain conditions which are included in the third group [8]. Despite the abundance of ILs in the literature, this article focuses on summarizing the role of ILs in NAFLD, specifically IL-1, IL-2, IL-3, IL-4, IL-5, IL-6, IL-7, IL-8, IL-,9 and IL-10. Notably, these ILs have not been collectively summarized and reported in previous studies, as depicted in Table 1. Figure 1 shows the mechanisms of action of cytokines.

## 2. Pathophysiological Aspects of Interleukins in NAFLD

The pathogenesis of NAFLD is complex and involves multiple factors, including genetics, lifestyle, and inflammation.

It is widely accepted that oxidative stress and inflammation represent key factors of NAFLD pathogenesis [3]. These processes are also the proposed underlying pathophysiological mechanism of the majority of disorders largely associated with NAFLD, such as obesity, insulin resistance, dyslipidemia, metabolic syndrome, and diabetes.

Visceral adipose tissue secretes a wide spectrum of adipokines and cytokines, compromising insulin sensitivity and leading to increased free fatty acids (FFA) hepatic flux, lipogenesis, liver fat peroxidation, i.e., oxidative stress, and inducing hepatocytes dysfunction and injury.

Insulin resistance is the central point in the development of NAFLD [4]. Namely, obesity-associated chronic low-grade systemic inflammation is a typical feature of NAFLD, increasing the levels of inflammatory cytokines, such as IL-6 and tumor necrosis factor-alpha (TNF-α) as the most studied, which may contribute to systemic inflammatory state and influence insulin signaling pathways, thus leading to a consequent insulin-resistant state. Insulin resistance promotes increased lipolysis of triglycerides (TG) in adipose tissue, with an excess of free fatty acids (FFA) that reach the liver and enhance oxidative phosphorylation, leading to an increase in reactive oxygen species production and liver fat peroxidation, i.e., oxidative stress. Increased cellular uptake of FFA without any subsequent β-oxidation contributes to the increased lipogenesis, as well as TG storage, inducing hepatic steatosis [5].

White adipose tissue releases adipokines and cytokines, thus leading to dysregulation of the expression of many ILs that are contributing factors to chronic inflammation.

ILs, which are signaling proteins implicated in the immune response, can play a role in the pathogenesis of NAFLD. ILs are key mediators of inflammation, and chronic inflammation is a hallmark of NAFLD. In the context of NAFLD, inflammation can be triggered by the accumulation of fat in the liver (steatosis). This inflammation can lead to a more severe form of NAFLD named NASH, which is characterized by liver cell injury and inflammation. Inflammatory cytokines have been implicated as crucial contributors to the development and progression of NAFLD, although their specific role remains inconclusive.

A recent meta-analysis that included 36,074 NAFLD patients and 47,052 controls examining a total of 19 pro-inflammatory cytokines revealed significant correlations for IL-1β, IL-6, C-reactive protein (CRP), and TNF-α with NAFLD. Conversely, no significant correlations were observed for IL-2, IL-4, IL-5, IL-7, IL-8, IL-10, and IL-12, with NAFLD. These findings suggest that such inflammatory mediators may serve as potential biomarkers for NAFLD, offering insights into its etiology and providing avenues for early diagnosis and intervention [9].

Interleukin-1

Macrophages are the main source of IL-1, a cytokine that has strong inflammatory and immune-boosting effects. IL-1 is mostly produced during defensive reactions. Eleven structurally related proteins make up the superfamily of IL-1 in mammals, all of which are essential for either inducing or controlling inflammation. These proteins attach to certain receptors on target cells’ plasma membranes to cause their actions. Ten transmembrane proteins with identical structural characteristics make up the family of IL-1 receptors, which assemble into heterocomplexes. The cytokines belonging to the IL-1 family not only have innate immunological and inflammatory effects, but they also aid in the establishment of adaptive immunity in vertebrates [10].

The factors influencing why only certain individuals with NAFLD develop NASH remain unclear, and currently, no drugs are approved for this specific indication. Various T-cell populations, including T-regulatory, Th1, and Th17 cells, play a pivotal role in the pathogenesis of NAFLD, opening up possibilities for future therapeutics targeting IL-17. The NASH is accompanied by increased expression of pro-inflammatory mediators (e.g., IL-1β and TNF-α). Anakinra, a recombinant sort of IL-1Ra, has demonstrated favorable cardiometabolic effects in type 2 diabetic patients, showing improved hyperglycemia and beta-cell secretory function [11,12]. Studies indicate that signals mediated by bile acid farnesoid X receptor (FXR), including the enterohepatic hormone fibroblast growth factor 15/19, play a role in regulating glucose and TG metabolism in NASH [11]. Recent studies have shown the favorable impact of the FXR agonist obeticholic acid on insulin sensitivity, body weight, and liver histology in NASH patients. Several potential new therapeutic agents for NASH are currently in phase II clinical trials, demonstrating a growing focus on advancing treatment options for this condition [11].

Two cytokines that belong to the IL-1 family, i.e., IL-1α and IL-1β, regulate inflammation by binding to IL-1R1 and IL-1R2, whereas IL-1Ra has inhibitory roles on these pathways. IL-1R1 favors the inflammation process via the stimulation of signaling pathways nuclear factor-kappa B (NF-κB) and the MAP kinase (MPK), whereas IL-1R2 is a decoy receptor due to the lack of cytoplasmic domain [13].

Unlike IL-1α being biologically active as a precursor biomolecule, IL-1β is mostly related to the late stages of inflammation by recruitment of the macrophages and the inflammasome complexes are required for the activation of pro-IL-1β. Inflammasome complexes are made of NLR family pyrin domain-containing 3 (NLRP3), the effector cysteine protease caspase-1 and the adaptor protein apoptosis-associated speck-like protein. Other inflammasomes that stimulate caspase 1 are NLRC4, NLRP2, and NLRP1 [13].

While pathways governing the activation of IL-1 family cytokines play a pivotal role in NAFLD development, the precise mechanisms remain incompletely understood. Numerous investigations have concentrated on the inflammasome/caspase-1 pathway, establishing its significance as a primary instigator of inflammation in NAFLD. Nevertheless, this pathway alone does not exclusively drive the activation of pro-inflammatory cytokines. Neutrophil serine proteases (NSPs) have emerged as an alternative mechanism capable of activating cytokines, with recent studies highlighting their contribution to NAFLD. These findings have, for the first time, provided evidence supporting the involvement of this inflammasome-independent pathway in NAFLD [14].

The accumulation of TG and liver steatosis and further necrosis promote the IL-1α secretion. In addition, hepatic Kupffer Cells (KCs), i.e., hepatic macrophages, favor the upregulation of IL-1α expression and recruitment monocytes and neutrophils to the site of inflammation, further contributing to the exacerbation of inflammation and liver injury. Moreover, the additional hepatic accumulation of TG occurs due to the properties of IL-1α that upregulate de novo lipogenesis genes in hepatocytes. Thus, IL-1α instigates an inflammatory response, i.e., the key feature of NASH progression [12].

NAFLD is linked to both hepatic and systemic insulin resistance. The mechanisms and extent to which hepatic inflammation influences insulin sensitivity in NAFLD remain incompletely elucidated. Another study investigated the impact of hepatic IL-1 signaling using a new conditional knockout mouse model, shedding light on the liver-specific functions of this signaling pathway. Male mice with hepatocyte-specific knockout of IL-1 receptor type 1 (IL-1r1Hep−/−) and wild-type (WT) littermates were subjected to a high-fat, high-carbohydrate diet (HFD) for 12 weeks. Both genotypes exhibited an obese phenotype and macrovesicular hepatic steatosis. However, in HFD-fed IL-1r1Hep−/− mice, microvesicular steatosis and ballooning injury were less pronounced, and ALT levels were within the reference range. Notably, in spite of HFD feeding, IL-1r1Hep−/− mice maintained high insulin sensitivity, evidenced by lower insulin levels, HOMA-IR, improved glucose tolerance, and reduced adipose tissue inflammation vs. WT mice. It underscored the contribution of hepatocyte IL-1R1 to (extra)hepatic insulin resistance. It suggested that treatment for NAFLD, by improving hepatic inflammation and IL-1R1 signaling, could have beneficial effects on insulin sensitivity [15].

A recent study has shown that IL-18 but not IL-1 signaling is the key initiator for liver injury in NAFLD in mice. IL-18R-dependent signaling and NLRP3 activation may be modulators of early liver damage in NAFLD [16].

IL-1β plays a pivotal role in the pathogenesis of NASH, which is a chronic liver disease, and age-related systemic inflammation. IL-1β is implicated in cardio-metabolic decline and may contribute to hepatic oncogenic transformation, making it a potential therapeutic target for these conditions. The effects of an IL-1β targeting monoclonal antibody on the liver and heart in an aged mouse model of NAS were investigated. Male C57Bl/6J mice, aged 24 months, were fed either a control or choline-deficient (CDAA) diet and treated with either isotype control or anti-IL-1β monoclonal antibody for 8 weeks. Cardiac functions were evaluated using conventional and 2D speckle-tracking echocardiography. It demonstrated improved cardiac diastolic function in mice with NASH treated with the anti-IL-1β antibody. Although significant hepatic fibrosis developed in the CDAA-fed group, IL-1β inhibition only affected fibrosis at the transcriptomic level and did not mitigate other features of NASH. Hepatic inflammation remained unaffected by the IL-1β inhibitor. Despite intensive hepatocyte proliferation observed in CDAA-fed animals, this process was not influenced by IL-1β neutralization. Interestingly, IL-1β inhibition led to an increase in the hepatic expression of immune checkpoint molecules Pd-1 and Ctla4, while Pd-l1 expression increased in NASH. In summary, IL-1β inhibition showed a positive impact on cardiac diastolic function but did not ameliorate NASH features. Additionally, it led to an increase in the expression of hepatic immune checkpoint molecules, accompanied by hepatocellular proliferation related to NASH [17].

The extent of inflammation significantly impacts the long-term outcomes of liver diseases, influencing the progression of liver fibrosis, cirrhosis, and HCC. Particularly, pro-inflammatory cytokines such as IL-1 (α and β) and TNF-α play a central role in various stages of liver diseases. They mediate fundamental aspects, including acute phase protein synthesis, lipid metabolism, cholestasis, and the degree of fibrosis. Released mainly by mononuclear cells, these key cytokines exert their influence on all liver cell types and orchestrate the production of numerous other mediators relevant to chronic liver diseases. In addition, inflammatory cytokines crucially regulate the development of insulin resistance, a key component of NAFLD. Despite ongoing research, blocking these critical inflammatory mediators, particularly TNF-α, with specific antibodies has not yet proven successful in treating alcoholic steatohepatitis, a disorder primarily driven by cytokines. In summary, inflammatory cytokines persist locally and systemically in individuals with advanced fatty liver diseases, shaping the clinical phenotype and influencing various features of these disorders [18].

NAFLD stands as the prevailing chronic liver ailment globally, with a rising incidence. Its progressive form, NASH, poses the risk of advancing to end-stage liver disease. Despite the increasing prevalence, the intricate pathogenesis of NAFLD remains incompletely understood, and there exists no specific treatment, necessitating the exploration of effective and dependable therapeutic approaches. Recent investigations have highlighted the crucial involvement of the inflammasome at various stages of NAFLD pathogenesis. Specifically, toll-like receptors detect pathogen-associated molecular patterns induced by the gut–liver axis, triggering the formation of the NLRP3 (NLR family pyrin domain-containing protein 3) inflammasome. Stimulation of damage-associated molecular patterns also activates this inflammasome. Once activated, the inflammasome exhibits caspase-1 activity, resulting in the release of IL-1 and IL-18, and the formation of pores in the cell wall. This process extends the inflammatory response beyond the cell and induces inflammatory cell death (pyroptosis). The ensuing progression of the inflammatory cascade contributes to fibrosis. Recent findings suggest that the NLRP3 inflammasome could serve as a potential target for NASH treatment. The identification of specific NLRP3 inflammasome blockers in recent years, along with evidence supporting their positive effects in experimental models, bolsters the viability of this therapeutic approach [19].

The mechanisms leading to NASH have remained elusive, and the non-invasive diagnosis of NASH presents challenges. The mRNA expression of IL1RN in the liver and serum IL-1RA levels were linked with NASH. Lower circulatory IL-1RA levels and diminished IL1RN expression following obesity surgery was related to the amelioration of lobular inflammation, suggesting the potential use of IL-1RA as a non-invasive inflammatory marker for NASH [20].

NAFLD, affecting a significant portion of the global population and closely linked to hypertension (HT), involves complex interactions of pro-inflammatory and anti-inflammatory cytokines in its pathology. Zhelezniakova et al. [21] showed that IL-1β levels were elevated in the NAFLD and HT group compared to the isolated NAFLD group and control group. Conversely, IL-10 levels were lower in both NAFLD groups compared to the control group. Strong relationships were found between CRP, IL-10, and IL-1β levels in patients with NAFLD and HT. Inverse correlations were also observed between IL-10 and IL-1β in patients with NAFLD, both being suggested as potential biomarkers for NAFLD progression [21].

Hadinia et al. [22] reported remarkably higher IL-1β and IL-6 in NASH patients compared to both NAFLD and controls, thus indicating that the clinical data of the early stage of NAFLD are similar to healthy subjects, and the development of steatosis and inflammation occurs as a result of increased pro-inflammatory mediators in NASH patients [22].

İlyas et al. [23] indicated that serum IL-6, IL-1β, and TNFα levels did not differ between the NAFLD patients and the controls. However, the IL-8 level was higher in the NAFLD group, suggesting a potentially more active role in the NAFLD pathogenesis as compared to TNF-α, IL-6, and IL-1β [23].

Chakraborty et al. [24] showed a significant relationship between the level of 25(OH) vitamin D and NAFLD in the young population. Both 25(OH) Vitamin D deficiency and high levels of serum IL-1a were independently associated with the risk of developing NAFLD [24].

Pro-inflammatory cytokines such as IL-1, IL-6, and TNF-α play a major role in the pathogenesis of NAFLD. These cytokines are also crucial in the development of insulin resistance, a key factor in NAFLD pathogenesis. Target-based therapy is suggested as a potential avenue for future research [25,26].

Sterile inflammation is a widespread response of tissues to stress and injury, particularly evident in the liver. This inflammatory reaction is heightened during the development of metabolic syndrome and excessive alcohol consumption, leading to significant tissue damage. In this context, IL-1β, an inflammatory cytokine, plays a crucial role in initiating and perpetuating inflammation and tissue damage. The activation of IL-1β involves cytosolic machinery collectively known as the inflammasome, as well as proteases released by neutrophils. While macrophages are the primary drivers of the inflammatory response, other hepatic cells, including hepatocytes, stellate cells, and sinusoidal endothelial cells, also contribute significantly. Hepatocytes secrete pro- and anti-inflammatory mediators. The hepatic inflammatory response is controlled through various mechanisms, with epigenetic regulation playing a major role. Notably, studies have demonstrated the fast progression of NASH in the offspring of female mice maintained on a high-fat diet before conception. This highlighted the crucial role of epigenetic regulation, which was likely occurring in adults experiencing prolonged metabolic stress. Understanding the epigenetic regulation of liver inflammation is not only clinically significant but also holds the potential to unveil new pathways for therapeutic intervention in liver diseases [27].

Interleukin-2

IL-2 is a specific type of interleukin that plays a crucial role in inducing the proliferation of responsive T-cells. Additionally, it exerts its effects on certain B-cells by binding to specific receptors, acting as a growth factor and stimulant for antibody production. Notably, IL-2 is not just a drug; it is a natural component of the immune system, functioning as a cytokine or messenger protein that activates various elements of the immune response. In the context of medical applications, for the treatment of metastatic renal cell carcinoma (RCC), IL-2 is the only medication licensed in the US. Approvals for it have also been granted in several other nations. IL-2 works by boosting the proliferation of immune cells, as opposed to conventional chemotherapy that kills tumor cells directly. Immune cells that can directly attack cancer cells include T-cells and Natural Killer Cells (NK Cells), which are especially impacted by IL-2 [28].

In recent studies within the Non-alcoholic Steatohepatitis Clinical Research Network (NASH CRN), soluble interleukin-2 receptor alpha (IL2RA) levels were observed to increase with fibrosis severity in both adult and pediatric cohorts. An examination of IL2RA’s potential as a biomarker for identifying non-alcoholic fatty liver disease was spurred by this connection, especially in Asian patients who are severely obese and having bariatric surgery. The results demonstrated that the NASH group had considerably greater levels of IL2RA expression than the non-NASH group, especially in immunohistochemistry. Multivariate analysis identified IHC of IL2RA and ALT as independent factors associated with NASH, with IL2RA demonstrating a notable odds ratio. The area under the receiver operating curve for IL2RA IHC in predicting NASH further supported its potential as a diagnostic biomarker. The findings suggest that IL2RA was significantly linked to NASH in morbidly obese patients and may serve as a valuable biomarker for diagnosing this condition [29].

There are yet no trustworthy non-invasive indicators for fibrosis, hepatic ballooning, or inflammation in NAFLD. In order to learn more about the cellular mechanisms behind NASH and to find putative non-invasive discriminators of NAFLD severity and patterns, a study examined the association between plasma cytokine levels and histological characteristics of NAFLD. Children with liver biopsy-verified NAFLD who were enrolled in NASH Clinical Research Network trials were included in the research. It was shown that children with confirmed NASH and lobular inflammation had greater levels of total plasminogen activator inhibitor 1 (PAI-1) and activated PAI-1. In patients with stage 3–4 fibrosis and lobular inflammation, IL-8 levels were greater; in children with stage 3–4 fibrosis and portal inflammation, soluble IL-2 receptor alpha levels were higher. Multivariable analysis revealed that PAI-1 factors were associated with lobular inflammation, ballooning, Definite NASH, and Borderline/Definite NASH discrimination. Steatosis and fibrosis severity were associated with an increase in IL-8, fibrosis severity and portal inflammation were associated with an increase in soluble IL-2 receptor alpha, and fibrosis severity and portal inflammation were associated with a drop in IL-7. It was highly associated with specific NASH and activated plasminogen activator inhibitor 1. Indicators of substantial fibrosis included reduced levels of insulin-like growth factor 2 and increased levels of soluble IL-1 receptor I, soluble IL-2 receptor alpha, resistin, IL-8, and TNF-ɑ. According to their research, some plasma biomarkers may be useful for non-invasively classifying NAFLD patients and identifying possible targets for therapeutic intervention by being strongly correlated with the disease activity and degree of fibrosis in the condition [30].

NAFLD is very common among obese teenagers. In research examining the pro-inflammatory cytokines in obese children and adolescents with and without NAFLD (a mean age of almost 12 years), 42.2% were classified as obese and 57.7% as severely obese. Hepatic steatosis was identified in 62.2% of participants, with approximately 90% having grade I steatosis. Although no difference in IL-2 and IL-6 levels were observed between the NAFLD and control groups, other inflammatory markers, including TNF-α and CRP, were elevated in the studied sample and positively correlated with the BMI for age and sex. Interestingly, hepatic steatosis prevalence was high but not directly related to the degrees of obesity. These findings suggest that inflammatory markers are elevated in obese children and adolescents, regardless of the presence of hepatic steatosis, highlighting the complex interplay between obesity, inflammation, and NAFLD in the pediatric population [31].

Interleukin-4

IL-4, known as the “prototypic immunoregulatory cytokine,” exerts diverse effects on various target cells, showcasing its versatility typical of cytokines. Its influence extends to important domains including hematopoiesis, antibody synthesis, inflammation control, and effector T-cell response generation. Particularly activated hematopoietic cells, such as T cells, Fc epsilon R1+ mast cells, and basophils, produce this cytokine specifically. Potential differences in the immunological effects of IL-4 produced from T cells and Fc epsilon R1+ cells are suggested by the variable tissue distribution and access to diverse target cells. Understanding the cell-specific regulation of IL-4 expression becomes particularly relevant given its association with disease pathology. Notably, aberrant expression of IL-4 correlates with certain diseases, emphasizing the importance of comprehending the signals governing its expression in a cell-specific manner. Recent advancements have shed light on the signaling pathways triggered by both T-cell and Fc epsilon R1 receptors, elucidating the mechanisms that stimulate IL-4 gene expression [32].

In a study by Das et al., NAFLD patients had higher levels of pro-inflammatory cytokines such as TNF-α and IL-6, but lower levels of the anti-inflammatory cytokine IL-4 and unaltered levels of IL-10 [33].

In an animal model of NAFLD, the effects of low altitude and hypobaric hypoxia on pro- and anti-inflammatory cytokine levels, notably TNF-α and IL-4, were studied. After 35 days in the pressure chamber, pro- and anti-inflammatory cytokine activity dramatically rose in the experimental group, outpacing that of the low-altitude group by more than twice. On the seventieth day, at high altitude, there were no appreciable variations from the low-altitude group. After five weeks, animals in hypobaric hypoxic conditions with a fructose-enriched diet showed a more than 1.5-fold rise in the cytokine index (TNF-α/IL-4). However, after ten weeks at altitude, TNF-α/IL-4 decreased in comparison to the low-altitude group, which showed the opposite trend. Notably, alterations in lipid metabolism were statistically linked with IL-4 and TNF-α levels. According to the study’s findings, NAFLD in rats fed a fructose-enriched diet under hypobaric hypoxic circumstances causes more significant disruptions in the lipid spectrum and the system of pro- and anti-inflammatory cytokines [34].

Interleukin-5

The eosinophilic aspect of the Th2 immune response is where IL-5, a homodimeric cytokine, mostly functions. Eosinophil survival, differentiation, and chemotaxis are all greatly aided by it. Maturity eosinophils are activated by IL-5, which enables them to degranulate and fight helminth worms. Remarkably, IL-5 does not contribute to the formation of IgE. Nevertheless, it can affect mast cells, encouraging the release of histamine linked to allergies. Moreover, IL-5 triggers the proliferation and differentiation of B cells and cytotoxic T lymphocytes (CTLs), upregulates IL-2Rα on B cells, and improves IgA production. However, as IL-5−/− animals do not exhibit impairment in B or T cell responses, the in vivo importance of IL-5 for these activities remains somewhat ambiguous. Owing to eosinophils’ role in Th2-mediated allergy and asthmatic reactions, pharmaceutical interest in IL-5 has grown. The lung damage caused by eosinophil degranulation generated by IL-5 in a mouse model of asthma was shown to be greatly reduced in IL-5−/− mice. It is possible that a medication that targets IL-5 might successfully reduce asthma episodes linked to eosinophilia without unduly affecting other aspects of host health because IL-5 seems to be a less pleiotropic cytokine than others [35].

NAFLD lacks a proven cure, making it a growing worldwide health problem. An evaluation of the effects of two distinct antioxidants on NAFLD in male Wistar rats was the goal of another investigation. When compared to the control group, it showed a statistically significant rise in serum IL-5 levels in the NAFLD group. Nevertheless, compared to the NAFLD group, treatment with propolis and vitamin E dramatically reduced IL-5 levels (*p* < 0.05), with the propolis group seeing the greatest reduction (*p* < 0.05). On the other hand, the fatty liver group showed a substantial increase in STAT1 gene expression when compared to the control group (*p* < 0.05). Following antioxidant treatment, the expression of this gene notably decreased (*p* < 0.05). The results suggest that the use of propolis and vitamin E, both natural antioxidants, holds promise in NAFLD therapy. These antioxidants exhibit a therapeutic role by reducing inflammatory interleukin levels and suppressing the expression of genes associated with NAFLD development [36].

Interleukin-6

As a versatile cytokine, IL-6 plays a crucial part in host defense through a variety of immunological and hematological functions as well as a strong ability to initiate the acute phase response. Multiple myeloma, rheumatoid arthritis, Castleman’s disease, psoriasis, and post-menopausal osteoporosis are among the illnesses for which the overexpression of IL-6 has been connected to the pathophysiology. As such, therapeutic promise exists for the creation of specific antagonists that target the activity of IL-6. Along with IL-11, leukemia inhibitory factor, oncostatin M, cardiotrophin-1, and ciliary neurotrophic factor, IL-6 is a member of the cytokine family. As with other members of the family, IL-6 stimulates proliferation or differentiation via a receptor system that uses a particular receptor in conjunction with the common signaling component gp130. The authors propose and discuss a preliminary model for the IL-6 hexameric receptor–ligand complex, providing insights into the common mechanism of action of other cytokines in the IL-6 family. The objective of this investigation on IL-6 interactions is to establish a logical framework for regulating this cytokine’s effects for therapeutic purposes [37].

Another study elucidated the role of the inflammatory cytokine IL-6 in the pathogenesis of non-alcoholic steatohepatitis, a prevalent liver disease with poorly understood inflammatory mechanisms. For five weeks, a methionine and choline-deficient (MCD) diet was fed to both wild-type and IL-6−/− mice to develop steatohepatitis. The MCD diet caused both genotypes to lose weight, but IL-6−/− mice showed a less marked rise in blood alanine aminotransferase than wild-type animals, despite equal decreases in serum glucose, cholesterol, and triglyceride levels. Compared to their wild-type counterparts, mice lacking IL-6 showed reduced lobular inflammation while having a similar level of hepatic steatosis. Furthermore, in IL-6−/− mice, the liver gene expression of TGF-β and MCP-1 was significantly reduced, although PPAR-γ and F4/80 transcripts and proteins showed a more moderate drop. The MCD diet in both normal and mutant mice caused a comparable drop in the ratio of phosphatidylcholine to phosphatidylethanolamine, as shown by chromatographic examination of liver lipids. On the other hand, the diet caused a less notable rise in sphingomyelin and ceramide levels in IL-6−/− animals. These results, taken together, suggest that IL-6 shortage does not stop NASH from developing. However, IL-6 plays a crucial role in the associated liver inflammation, as evidenced by reduced lobular inflammation and attenuated expression of key inflammatory genes in IL-6-deficient mice subjected to the MCD diet [38].

Growing public health concerns are associated with type 2 diabetes and NAFLD, and little is known about their substantial correlation. One of the main pro-inflammatory cytokines, hepatic IL-6, is elevated in animal models of NAFLD, and chronic elevation in mice leads to systemic insulin resistance. However, nothing is known about the degree and clinical relevance of hepatic IL-6 expression in human NAFLD, or about the possible pathways that connect steatosis to elevated IL-6 production. It is shown that individuals with NASH had significantly higher levels of hepatic IL-6 expression than patients with simple steatosis or normal biopsies, indicating the existence of hepatic IL-6 expression in human NASH. The degree of inflammation and the stage of fibrosis were shown to positively correlate with the expression of IL-6 in hepatocytes. The degree of systemic insulin resistance and plasma IL-6 levels was significantly linked with liver IL-6 expression. Saturated fatty acids (FFAs) significantly increased the production of IL-6 messenger RNA (mRNA) and protein in investigations involving the culture of liver cells; this effect was not observed with mono- or polyunsaturated FFAs. These results imply that increased hepatic IL-6 production may be involved in systemic insulin resistance, diabetes, and NASH development [39].

A target-based Mendelian randomization was used to investigate the efficacy of interleukin-6 receptor (IL-6R) blockage, which has been licensed for inflammation-associated disorders, in the treatment of non-alcoholic fatty liver disease. The objective of a different investigation was to determine if NAFLD risk may be decreased by blocking the IL-6 signaling pathway by IL-6R blockage. A genetic proxy single nucleotide polymorphism (SNP) called rs2228145 was used to assess therapy results. This SNP was previously found to imitate the effects of IL-6R blockage. From non-FinnGen GWAS (1483 cases and 17,781 controls) and FinnGen GWAS (894 cases and 217,898 controls), genetic correlations between SNP rs2228145 (A > C) and NAFLD were discovered. A Wald ratio approach was used to quantify causal effects, which were then merged using a fixed-effects meta-analysis. SNP rs12048091 was used as an additional proxy in the sensitivity analysis. Positive control analysis provided evidence in favor of the hypothesis that SNP rs2228145 replicates the effects of IL-6R blocking by lowering the risk of coronary heart disease and rheumatoid arthritis. While not significant in the FinnGen consortium, the Mendelian randomization analysis in the non-FinnGen GWAS revealed that IL-6R blockage would raise the risk of NAFLD. The fixed-effects meta-analysis suggested that NAFLD risk might be decreased by blocking the IL-6 signaling pathway. The meta-analysis utilizing two genetic variants also revealed a comparable impact on NAFLD when SNP rs12048091 was included as a genetic instrument. Overall, the results of this Mendelian randomization research indicated that IL-6R blockage may raise the risk of non-alcoholic fatty liver disease by blocking the IL-6 signaling pathway. This suggests that IL-6R could protect against NAFLD [40].

Insulin resistance is a feature of NAFLD, which is frequently linked to diabetes and obesity. Adipose tissue is the primary source of cytokines and adipocytokines, which are essential in coordinating inflammatory processes throughout the body and controlling a number of processes, including immune response, inflammation, and metabolism, including IR. The pathogenesis of several features of NAFLD is influenced by pro-inflammatory cytokines, specifically TNF-α and IL-6. Genetic modification and overnutrition have proven the role of TNF-α in fatty liver disorders in both humans and animals. Studies conducted on animals also demonstrate that fatty liver disease and IR can be improved by blocking TNF-α activity. There is a high link observed between the blood levels of IL-6, which is generated by different cells such as adipocytes, and the presence of inflammation. It has been determined that IL-6 generated from adipose tissue regulates hepatic IR via an upregulating suppressor of cytokine signaling 3 (SOCS3). In vitro and experimental animal studies have demonstrated the ability of adiponectin, an adipocytokine with strong TNF-α-neutralizing and anti-inflammatory capabilities, to mitigate inflammation and IR. By suppressing TNF-α production and inducing other anti-inflammatory cytokines like IL-10 or IL-1 receptor antagonists, it has anti-inflammatory effects. Thus, the development of NAFLD is thought to be largely dependent on the delicate balance between pro- and anti-inflammatory cytokines/adipocytokines in hepatic and systemic insulin action [41].

The development of hepatic steatosis and its progression to NASH are mostly attributed to inflammatory mechanisms. The well-known pro-inflammatory cytokine IL-6 aids in liver regeneration and shields the organ from harm. It is still unclear, nevertheless, exactly what part IL-6/Glycoprotein130 (GP130) plays in NASH. In a mouse model of non-alcoholic steatohepatitis, Yamaguchi et al.’s study sought to ascertain if inhibiting IL-6/GP130 signaling may stop the disease from progressing. For eight weeks, male C57/BL6 mice that were six weeks old were given either a chow control or a methionine choline-deficient (MCD) diet. While the remaining MCD and chow-fed mice got 15 mg/kg rat IgG as a control, half of the mice on the MCD diet received intraperitoneal injections twice a week at a dose of 15 mg/kg rat anti-mouse IL-6 receptor antibody (MR16-1). Evaluation criteria encompassed hepatic steatosis, damage, fibrosis, apoptosis, lipid peroxidation/oxidant stress indicators, and gene expressions linked to IL-6. The administration of MR16-1 to mice on an MCD diet led to a reduction in the activities of the signal transducer and activator of transcription 3 as well as the expression of the suppressor of cytokine signaling 3. While increasing sterol regulatory element-binding protein 1 and decreasing peroxisome proliferator-activated receptor-α expression, this treatment also increased intrahepatic lipid accumulation. However, it improved elevated plasma ALT levels with decreased levels of plasma-free fatty acid, lipid peroxidation/oxidant stress, and hepatic apoptosis. Hepatic steatosis generated by the MCD diet was exacerbated but liver damage was reduced when MR16-1 blocked IL-6/GP130 signaling. These results indicate that hepatic IL-6 signaling may increase liver inflammation but may also have a preventive effect against the development of hepatic steatosis [42].

The rs738409 C > G single nucleotide polymorphism (SNP) has been highly connected with NAFLD, and other genetic polymorphisms have also been linked to susceptibility to or protection against NAFLD. The I148M variation of patatin-like phospholipase domain-containing protein 3 (PNPLA3) is the product of this SNP. Still unclear, though, are the underlying processes of this connection. In order to explore these pathways, scientists created a multicellular liver culture obtained from human pluripotent stem cells (hPSCs), which included macrophages, hepatic stellate cells, and hPSC-derived hepatocytes. By subjecting the cells to a lipotoxic environment that mirrored the levels of disease risk factors in circulation, the hPSC-derived liver culture effectively represented NAFLD. After that, two isogenic liver cultures were made, with the exception of one rs738049 SNP difference, and the development of the NAFLD phenotype was compared. It proved that, under lipotoxic environments, the I148M mutation accelerated the development of NAFLD phenotypes. These variations were linked to increased liver culture IL-6/signal transducer and activator of transcription 3 (STAT3) activity, which is in line with transcriptome information from liver biopsies of people who had the rs738409 SNP. In wild-type liver cells, increasing IL-6/STAT3 activity led to an increase in NAFLD development, whereas decreasing it reduced the I148M-mediated vulnerability to NAFLD. It suggested that increased IL-6/STAT3 activity and I148M-mediated vulnerability to NAFLD could be causally related. The heightened activity in liver cultures with the rs738409 SNP was ascribed to enhanced NF-κB activation [43].

Soluble IL-6 receptor blood levels were assessed in a study evaluating patients with various clinical and morphological manifestations of NAFLD and healthy donors. The correlation between the soluble IL-6 receptor and IL-6 content, IL6 gene mRNA expression, and several indicators of apoptosis in hepatocytes and peripheral blood leukocytes was assessed. It was determined for the first time that alterations in the blood’s concentration of soluble IL-6 receptors were linked to the advancement of NAFLD. When compared to early stages of NAFLD development, such as liver steatosis and NASH of weak and moderate activity, the blood concentration of the soluble IL-6 receptor was significantly lower in individuals with high activity of NASH and liver cirrhosis. It is thought that this drop in the concentration of the soluble IL-6 receptor may be a novel diagnostic sign for differentiating between NASH with high activity and NASH with weak and moderate activity. Additionally, it demonstrated a strong association between alterations in soluble IL-6 receptor levels and hepatocyte and peripheral blood leukocyte death. This implies that, in the setting of NAFLD development, there may be a connection between soluble IL-6 receptor levels and apoptosis in both peripheral blood leukocytes and hepatocytes [44].

In individuals living with diabetes, there is often a co-occurrence of NAFLD. The role of IL-6 in both conditions, particularly its interaction with membrane-bound (classical) and circulating (trans-signaling) soluble receptors, was investigated. An investigation of the potential correlation between the severity of fatty liver disease and changes in the secretion patterns of IL-6 trans-signaling co-receptors in NAFLD linked with diabetes was the focus of separate research. Human hepatocyte, stellate, and monocyte cell lines were used in the study to examine secretion patterns. Two patient groups were examined for associations with liver pathology: 1) those with class 3 obesity and 2) those with biopsy-confirmed steatohepatitis. The following are some of the study’s main conclusions: Stellate cells exposed to high glucose and palmitate secreted more IL-6 and soluble gp130 (sgp130). Diabetes patients had increased levels of circulating IL-6 and trans-signaling co-receptors, and plasma sgp130 levels positively linked with HbA1c in both patient groups. Patients with an F4 fibrosis stage were shown to have greater levels of liver stiffness in correlation with plasma sgp130. Reduced production of soluble IL-6 receptor (sIL-6R) was linked to monocyte activation. In people with concurrent diabetes, the existence of hyperglycemia and hyperlipidemia may have a direct effect on IL-6 trans-signaling, which may enhance the severity of NAFLD [45].

Obesity and IR are two characteristics that are closely linked to NAFLD. The significance of IL-6 assessment as a possible predictor of NASH and a tool for early intervention to avoid disease development is highlighted by the high prevalence of NAFLD, a disorder that shares many characteristics with the metabolic syndrome [46,47]. IL-6 was shown as a predictor of NAFLD among individuals with Insulin Resistance Syndrome (IRS) [48]. The pro-inflammatory IL-6 has a contentious function in the setting of hepatic steatosis, a risk factor for the development of NAFLD. Using a rat model fed with a choline-deficient (CD) diet, which causes hepatic steatosis, a study examined the effects of IL-6 on the lipid content of the liver and mitochondria as well as cell death. Phosphatidylcholine (PC), phosphatidylethanolamine (PE), and the membrane integrity measure PC: PE ratio were all reduced in the choline-deficient diet, whereas liver triglycerides and cholesterol increased. The hepatocytes’ susceptibility to apoptosis, systemic IL-6, and IL-6 receptor expression were likewise increased by this diet. The electron transport chain was disrupted and the phospholipid composition changed, with increased triglycerides, cardiolipin, PC, and PC:PE ratio, as demonstrated by mitochondria in the liver of the CD rat. Treatment with IL-6 attenuated the lipid disruption in mitochondria caused by the CD diet in primary hepatocyte cultures, but it did not stop TNF-α-induced cell death. On the other hand, PC supplementation stopped TNF-α-induced caspase-3 activity, cytochrome c release, and DNA fragmentation in both CD and control hepatocytes. Hepatocytes derived from steatotic mice showed improved mitochondrial lipid imbalance in response to IL-6. Furthermore, PC was shown to be a survival agent with the ability to reverse a number of TNF-α-induced reactions that might be involved in necrosis and steatosis [49]. Another study showed that IL-6 only demonstrated an elevation in NASH patients, but TNF-α significantly increased in all NAFLD categories, i.e., simple and moderate steatosis and NASH [50].

The presence of low-grade chronic inflammation (i.e., higher IL-6 levels) is also confirmed in obese children with NAFLD as compared with healthy controls. On the other hand, IL-10 may act as a protective factor against the development of NAFLD being higher in a subgroup of obese children with steatohepatitis as compared to obese children free of NAFLD or those with simple fatty liver [51].

Interleukin-7

Originating in stromal cells, IL-7 is vital for providing lymphoid cells with vital signals in the early stages of development. Lymphoid progenitors have been shown to respond to IL-7 in two different ways: (1) by a trophic impact, and (2) by supporting V(D)J recombination. The IL-7Rα and γc are the two chains that make up the IL-7 receptor. Many kinase classes, including the Janus and Src families as well as PI3-kinase, are rapidly activated during receptor crosslinking. Following this, a number of transcription factors, including AP-1, NFAT, c-myc, and STATs, are active. Still unknown, though, is whether any of the previously discovered routes lead to the trophic or V(D)J destinations. Protecting lymphoid progenitors from a process that resembles apoptosis is the trophic response to IL-7. Other IL-7-induced processes are probably implicated in the trophic response, even though Bcl-2 activation by IL-7 contributes to this protection. Rag proteins that cleave the target locus are produced in greater quantities in the context of the V(D)J response to IL-7, which serves as part of the process. Furthermore, by chromatin remodeling, IL-7 increases a target locus’s susceptibility to cleavage, which aids in the V (D)J response [52].

In order to learn more about the cellular mechanisms driving NASH and to find putative non-invasive discriminators of NAFLD severity and pattern, the association between plasma cytokine levels and different histological characteristics of NAFLD was examined in young populations with “Definite NASH”, “Borderline NASH”, and NAFLD but not NASH. The soluble IL-2 receptor alpha increased with the degree of fibrosis and portal inflammation, whereas IL-8 increased with the severity of steatosis and fibrosis. IL-7 lowered with the degree of fibrosis and portal inflammation. To clarify these markers’ function in NAFLD and assess their potential as non-invasive disease severity discriminators, further focused research is needed [53].

Interleukin-8

Produced by a variety of tissue and blood cells, IL-8 is a chemotactic cytokine that only targets neutrophils and has minimal impact on other blood cells. Its role in attracting and activating neutrophils in inflammatory regions has been recognized, particularly in conditions like periodontal disease where neutrophils play a crucial role. Neutrophils are a major population of immune cells in periodontitis, and their dysfunctions can lead to rapid loss of periodontal tissue. Migration, the release of granule enzymes, and other intra- and extracellular alterations are the reactions of neutrophils to IL-8. When activated, neutrophil enzymes are produced, which effectively break down the components of connective tissue. With structural similarities to other short chemotactic peptides and DNA sequence traits indicating potential shared regulation mechanisms with other cytokines, IL-8 is a member of the Interleukin-8 supergene family. When IL-8 is applied topically in vivo, it causes localized exudation as well as a large, sustained neutrophil buildup. Although IL-8 is involved in the larger cytokine network, its main pathophysiological function is to affect neutrophil behavior [54].

A recent study has shown that women with morbid obesity had higher levels of IL-1β, IL-8, IL-10, and TNF-α. In such women, increased levels of IL-8 were linked to the diagnosis of NASH. Moreover, an association was found between higher circulating levels of IL-8 and the hepatic expression of toll-like receptor 2 (TLR2), suggesting a potential role of TLR2 in the NASH pathogenesis, in relation with IL-8 [55].

Genetic polymorphisms are implicated in the pathophysiology of NASH, prompting an investigation into IL-6 and IL-8 gene polymorphisms in NASH pathogenesis. However, Cengiz et al. showed that polymorphisms in the IL-6 and IL-8 genes do not appear to have a role in the pathophysiology of NASH or liver fibrosis [56].

Birerdinc et al. [57], in a cohort of 241 morbidly obese patients, showed that IL-8 positively correlates with several cytokines (e.g., IL-10, IL-12, IL-13, IL-17, and TNF-α). Liver damage indicators (e.g., such as ballooning, bridging, infiltrating Kupffer cells, polymorphonuclear neutrophils, pericellular fibrosis, portal fibrosis, and portal inflammation) showed positive correlations with IL-8, suggesting that IL-8 plays a crucial role in systemic inflammation progression and, more importantly, contributes significantly to the escalating severity of liver disease in NAFLD [57].

Another study examined the hepatic overexpression of IL-8, a key chemokine responsible for neutrophil recruitment in humans, in an attempt to better understand the function of neutrophil infiltration in the transition from fatty liver to NASH. Mice fed a high-fat diet (HFD) for three months developed fatty livers without concurrent inflammation or fibrosis. Adenovirus overexpressing human IL-8 was then used to infect mice for a further two weeks, which elevated IL8 levels, neutrophil infiltration, and liver damage. Mechanistically, the increase of NADPH oxidase 2 complex components implicated in the neutrophil oxidative burst was linked to IL-8-induced liver damage. The development of macrophages and the increase of pro-inflammatory cytokines and chemokine expression were promoted by IL-8-stimulated infiltrating neutrophils. Crucially, in HFD-fed animals, IL-8 overexpression increased fibrosis-related variables such as collagen deposition and hepatic stellate cell activation. In conclusion, it was shown that in HFD-fed mice, hepatic overexpression of human IL-8 stimulates neutrophil infiltration and accelerates the transition from fatty liver to NASH [58].

The relationships between the histopathological features of NAFLD and the levels of several adipokines and circulating inflammatory markers (i.e., IL-6, IL-8, TNF-α, and CRP) were examined in a study involving 19 obese women undergoing bariatric surgery. When compared to the group without fibrosis, the study group of obese women with liver fibrosis showed noticeably higher levels of IL-8. When applied to similar populations, these markers may yield useful information for assessing NAFLD [59].

One of the most important indicators of liver-related morbidity and death in NAFLD patients is the degree of hepatic fibrosis. In order to identify the cytokines linked to liver fibrosis, baseline blood samples from 97 individuals with biopsy-proven NAFLD were analyzed. Patients were classified according to the degree of hepatic fibrosis they had, and it was discovered IL-8 was linked to liver fibrosis. When compared to recognized indicators of hepatic fibrosis, IL-8 also shown a strong predictive ability for greater fibrotic liver damage. IL-8 gene expression was shown to be elevated and differently expressed in patients with advanced hepatic fibrosis, according to hepatic gene expression research performed on 72 more NAFLD patients. Serum levels of IL-8 may indicate elevated gene expression during NAFLD-related liver fibrosis. To sum up, cytokines like IL-8 may be used as diagnostic tools for advanced liver fibrosis in NAFLD, offering new avenues for the development of antifibrotic treatments. The argument for these analytes as suitable surrogate blood biomarkers for the severity of hepatic fibrosis is strengthened by the observed association between elevated serum levels and related hepatic gene expression [60].

Interleukin-9

Many different types of cells, including mast cells, NKT cells, Th2, Th17, Treg, ILC2, and Th9 cells, release IL-9, a versatile cytokine with pleiotropic effects. It is thought that Th9 cells in particular are the main CD4+ T cells that produce IL-9. The cytokine affects a variety of organs and cell types. It has been determined that it is mostly involved in immunological responses against parasites as well as the pathophysiology of allergy illnesses such as asthma and bronchial hyper-reactivity. However, it also contributes to the development of hematologic neoplasias, which in humans includes Hodgkin’s lymphoma. It is interesting to note that IL-9 has anticancer effects on solid cancers like melanoma [61].

The influence of taurine (Tau) on NAFLD prevention and therapy was examined in the study using a rat model. Rats on a HFD showed higher blood levels of LDL, cholesterol, triglycerides, ALT, and AST than the normal control and NP groups. TG and serum AST were found to be reduced in the Tau-prevention and Tau-treatment groups compared to the HFD rats (*p* < 0.05). Histological analysis of the livers of HFD rats showed extensive fatty degeneration and infiltration of inflammatory cells, in addition to damaged or missing villi, exfoliated epithelium, and infiltration of inflammatory cells in the ileal mucosa. In the liver and ileal mucosa of HFD rats, TGF-β, IL-9, and their mRNA levels were considerably higher compared to the normal control and NP groups. These levels were considerably lower in the Tau-prevention and Tau-treatment groups compared to the HFD rats. According to the research, taurine may be able to prevent or treat ileal mucosa and liver damage in rats suffering from NAFLD by downregulating the production of TGF-β and IL-9 [62].

Interleukin-10

As an anti-inflammatory cytokine, IL-10 is known to be important in regulating the immune response. IL-10 functions in the setting of infections by suppressing the function of macrophages, natural killer (NK) cells, and Th1 cells—all of which are necessary for efficient pathogen clearance but may potentially exacerbate tissue damage. As thus, IL-10 serves two purposes by restricting the removal of pathogens and reducing immunopathology. IL-10 is produced by a variety of cell types, and its primary cellular source might change depending on the tissue or infection stage [63].

Recent research has shed light on the significant role of IL-10 not only in autoimmune diseases, inflammatory conditions, and cancers but also as a key regulatory factor in NAFLD pathogenesis [64].

Insulin resistance is a hallmark of NAFLD, which is linked to obesity, diabetes, and poor liver function in the Western world. Pro-inflammatory cytokines like TNF-ɑ are among the mediators that are generated by immune system cells or adipocytes to control IR. It has been established how important TNF-ɑ is in fatty liver illnesses in humans and animals that are brought on by genetic modification or overnutrition. It has been demonstrated that IR and fatty liver disease in animals can be improved by neutralizing TNF-ɑ activity. In vitro and experimental animal investigations demonstrate that the anti-inflammatory adipokine adiponectin efficiently neutralizes TNF-ɑ and counteracts inflammation and IR. Suppression of TNF-ɑ production and induction of anti-inflammatory cytokines such as IL-10 or IL-1 receptor antagonists are two of adiponectin’s anti-inflammatory actions. Thus, the development of NAFLD and the action of insulin in the liver and systemic circulation seem to depend on the balance between different immune system or adipose tissue mediators [65].

About 25% of adults have NAFLD, which is strongly associated with HT. It is still necessary to search for non-invasive NAFLD indicators. In a different study, the diagnostic utility of IL-10 and IL-1β in conjunction with HT for the diagnosis of NAFLD was assessed. Levels of IL-10 and IL-1β significantly changed over the course of both comorbid NAFLD with HT and isolated NAFLD. In individuals with NAFLD and HT, considerably more dramatic deviations of these markers were linked to greater HT stage and blood pressure grade, elevated BMI, and elevated CRP levels. Based on the results acquired, IL-10 and IL-1β may be taken into consideration as biomarkers of the severity of NAFLD [66].

In NASH, characterized by fatty liver and inflammation, the role of IL-10, a crucial anti-inflammatory cytokine, was explored in mouse models using a liquid diet containing 5% ethanol for 4 weeks or a 12-week HFD. The study involved IL-10 knockout (IL-10(−/−)) mice and other genetically modified strains. Contrary to the hypothesis, IL-10(−/−) mice exhibited a more pronounced liver inflammatory response with elevated IL-6 levels and hepatic signal transducer and activator of transcription 3 (STAT3) activation. In contrast to wild-type mice, these animals had reduced steatosis and hepatocellular damage following alcohol or HFD feeding. In IL-10(−/−) animals, further deletions of IL-6 or hepatic STAT3 increased the inflammatory response in the liver but reversed steatosis and hepatocellular damage. Moreover, the investigation demonstrated that sterol regulatory element-binding protein 1 as well as important downstream lipogenic proteins and enzymes involved in fatty acid synthesis were downregulated in IL-10(−/−) mice. In contrast, phosphorylated adenosine monophosphate-activated protein kinase and its downstream targets, such as phosphorylated acetyl-coenzyme A carboxylase and carnitine palmitoyltransferase 1, were found at higher levels in the livers of IL-10(−/−) animals. Double knockout mice of IL-10(−/−) IL-6(−/−) or IL-10(−/−) STAT3 (Hep−/−) were able to rectify the dysregulations shown in IL-10(−/−) animals. The study found that although IL-10(−/−) mice were resistant to hepatocellular injury and steatosis brought on by ethanol or high-fat diet, they were susceptible to an inflammatory response in the liver. The resistance to steatosis was linked to an increase in inflammation-associated hepatic IL-6/STAT3 activation, which in turn caused the liver to downregulate lipogenic genes and upregulate genes linked to fatty acid oxidation [67].

## 3. Current Treatment Options for NAFLD and NASH

Lifestyle intervention, healthy dietary patterns, and weight loss programs are the first treatment options for NAFLD. In case such interventions do not give expected results, other treatment options that are among the most promising are antidiabetic medications, agonist of farnesoid X receptor (FXR), or even bariatric surgery [68].

- Antidiabetic treatment

Given the tight relationship between IR, type 2 diabetes, and NAFLD, several antihyperglycemics were used as a treatment option for NAFLD/NASH, especially for patients who are overweight/obese [69,70]. Despite weak evidence related to the beneficial effect on liver fat and fibrosis, metformin is the first treatment option for type 2 diabetes and NAFLD [70]. If the glycemic control is not achieved with metformin, other treatment options are as follows: thiazolidinediones, sodium-glucose cotransporter 2 (SGLT2) inhibitors, and glucagon-like peptide 1 (GLP1) receptor antagonists [69,70].

Thiazolidinediones (e.g., rosiglitazone and pioglitazone) are peroxisome proliferator-activated receptor (PPARγ) agonists acting to enhance insulin sensitivity and diminish hepatic steatosis [69]. Pioglitazone was shown to reduce lobular inflammation and hepatic steatosis and even improved liver fibrosis, as confirmed by a recent meta-analysis that included five randomized controlled trials with pioglitazone in patients with NAFLD, thus being regarded as the current treatment option for patients with T2DM and NAFLD [70]. However, rosiglitazone was related to adverse cardiovascular outcomes [70].

SGLT2 inhibitors: (e.g., canagliflozin, empagliflozin, and dapagliflozin) exhibit inhibitory properties on SGLT-2 proteins in the renal tubules, which are accountable for the reabsorption of glucose, thus leading to an enhancement of the urinary loss of glucose. Recent studies have shown steatosis improvement by SGLT-2 inhibitors in patients with NAFLD [70].

GLP1 receptor antagonists (e.g., liraglutide, semaglutide, exenatide, dulaglutide) act as inducers of pancreatic β cells to secrete insulin, have beneficial effects on NAFLD, mostly due to systemic alterations in the metabolism rather than a direct effect on the liver itself [69]. NAFLD patients exhibit insufficient GLP-1 secretion. Significant improvement of NASH histology in almost 40% of patients with biopsy-proven NASH on liraglutide was confirmed, accompanied by a significant number of patients with weight loss [70]. A recent study using a NAFLD mouse model has shown that semaglutide reduced pro-inflammatory cytokines (IL-1b, TNF-a, IL-6,), as well as the mice liver and body weight, and liver steatosis [71]. GLP-1 agonists are also regarded to slow down chronic kidney disease progression and reduce cardiovascular and all-cause mortality. However, the specific use of GLP-1 agonists to treat NAFLD is unlicensed, and future studies are needed to unravel their utility in the NAFLD treatment [70].

Other antidiabetic drugs that include dipeptidyl peptidase 4 (DPP4) inhibitors, sulfonylurea agents, and insulin show no benefit for the NAFLD, exhibit significant unfavorable properties (worsening of sarcopenia or weight gain), or they are discouraged in NAFLD treatment (e.g., insulin) [70].

- IL-1 inhibitors

A few experimental studies reported an important role for IL-1 type cytokines in NAFLD, showing that blocking IL-1β has favorable properties on some aspects of metabolic disorders. These treatment options that target specific IL1 cytokines by different strategies (e.g., small molecules, monoclonal antibodies) are promising therapeutics in the new era of drugs development for inflammatory diseases [72].

In the CANTOS trial (double-blind randomized) that encompassed a total of 10,061 patients with high cardiovascular risk, a human monoclonal anti-IL1β antibody, i.e., canakinumab, ameliorated metabolic inflammation and reduced cardiovascular events during a follow-up period of about 3.7 years [73].

Although the results on hepatic steatosis are not available from this trial, considering the fact that canakinumab administration in patients with diabetes ameliorated hyperglycemia over a period of 1 year, it is assumed as a promising treatment option for NAFLD [74].

However, some cytokines share the same receptor and via its activation or inhibition, several inflammatory pathways mediated by those monoclonal antibodies or small molecules can be influenced. In line with this, IL-1β, IL-1α, IL-36, and IL-33 share IL1RAcP. Additionally, some receptors/cytokines have pro-inflammatory properties (IL-1β, IL-1α, and IL18), and others have anti-inflammatory action (IL-37), thus facilitating to act in two opposite ways on inflammatory processes in liver disease, depending on whether antagonist or agonist actions are stimulated [75].

Importantly, in addition to activation of the classical NLRP3/inflammatory caspase-1 cytokine pathway, the activation of IL-1β and IL-18 also included neutrophil serine proteases (NSPs), which might in part elucidate why inhibition of NLRP1 and NLRP3 inflammasomes exhibited low potency [75,76].

Therefore, the discovery of therapeutics should focus on the target of the majority of mediators related to the stimulation of signaling pathways of several pro-inflammatory cytokines, such as alpha-1 antitrypsin, an inhibitor of NSPs which has protective effects on NAFLD progression in animal studies [77,78].

However, the evidence of the efficacy of the administration of IL-1 inhibitors in humans is limited. Moreover, the functions of all IL-1 family members are not completely enlightened [75].

## 4. Conclusions

The development of NAFLD is significantly influenced by ILs. The exact mechanisms by which ILs contribute to NAFLD may vary, and ongoing research is aimed at understanding the specific roles of different ILs in the pathogenesis of this condition. The lack of clinical studies related to the potential therapeutic properties of IL-1 inhibitors currently does not allow us to conclude their validity as a therapeutic option, although preclinical studies show promising results. Further studies are needed to elucidate their beneficial properties in NAFLD treatment.

## Figures and Tables

**Table 1:** Summary of pathophysiological aspects of interleukins in NAFLD.

ILs	Pathophysiological Aspects in NAFLD
IL-1	The manufacture of extracellular matrix proteins, hepatocyte malfunction, necrosis, apoptosis, and the release of pro-inflammatory cytokines such TNF-α and IL-1, which ultimately result in cirrhosis and fibrosis [12]. IL-18 but not IL-1 signaling is the key initiator for liver injury in NAFLD in mice. IL-18R-dependent signaling and NLRP3 activation may be modulators of early liver damage in NAFLD [16]. Whereas Pd-l1 expression increased in NASH, IL-1β suppression boosted the hepatic expression of immunological checkpoint molecules Pd-1 and Ctla4 [17]. This inflammasome is also activated by stimulation of damage-associated molecular patterns. Following activation, the inflammasome displays caspase-1 activity, which causes the cell wall to become porous and releases IL-1 and IL-18. This procedure causes inflammatory cell death (pyroptosis) and expands the inflammatory response outside of the cell. Fibrosis is a result of the inflammatory cascade that follows [19]. The degree of lobular inflammation in the liver and NASH were related to blood levels of IL-1RA and hepatic mRNA expression of IL1RN [20]. IL-1β and IL-10 might be biomarkers for the advancement of NAFLD in both its independent course and in conjunction with HT [21]. The development of steatosis and inflammation in NASH patients is due to an excess of pro-inflammatory factors (IL-1beta, IL-6, and TNF-ɑ), and the early stage of NAFLD is comparable to that of healthy persons [22]. In this context, IL-1β, an inflammatory cytokine, plays a crucial role in initiating and perpetuating inflammation and tissue damage. The activation of IL-1β involves cytosolic machinery collectively known as the inflammasome, as well as proteases released by neutrophils. While macrophages are the primary drivers of the inflammatory response, other hepatic cells, including hepatocytes, stellate cells, and sinusoidal endothelial cells, also contribute significantly. Acute phase reactants, which are released by hepatocytes and have both pro- and anti-inflammatory properties, are a primary source of chemicals linked to damage. However, the overall effect seems to be context-dependent. On the other hand, stellate cells can control the development of regulatory T cells by producing all-trans retinoic acid and transforming growth factor (TGF)-β [27].
IL-2	The NASH group’s IL2RA expression was noticeably greater than that of the non-NASH group [29]. Infants with portal inflammation and stage 3–4 fibrosis had greater levels of soluble IL-2 receptor alpha [30].
IL-3	Undetermined
IL-4	Disruptions in lipid metabolism were significantly associated with levels of TNF-α and IL-4 [34].
IL-5	Serum IL-5 levels in NAFLD group were significantly higher than those of the control group [36].
IL-6	TNF-α, IL-6, and other pro-inflammatory cytokines are important players in the pathophysiology of NAFLD. These cytokines are also essential for the emergence of insulin resistance, which is a major component in the pathophysiology of NAFLD [25]. Furthermore, TGF-β and MCP-1 liver gene expression was significantly reduced in IL-6−/− animals, but PPAR-γ and F4/80 transcripts and proteins showed a more moderate decrease. However, in IL-6-deficient animals fed the MCD diet, lobular inflammation was decreased and the expression of important inflammatory genes was inhibited, indicating that IL-6 is essential for the related liver inflammation [38]. In animal models of NAFLD, hepatic IL-6, a major pro-inflammatory cytokine, is upregulated, and sustained elevation in mice results in systemic insulin resistance. These findings suggest that elevated hepatic IL-6 production may play a crucial role in the development of NASH, as well as in systemic insulin resistance and diabetes [39]. Pro-inflammatory cytokines, including TNF-α and IL-6, have a pivotal role in the pathogenesis of several features of non-alcoholic fatty liver disease in humans [41]. Hepatic steatosis generated by the MCD diet was exacerbated when MR16-1 blocked IL-6/GP130 signaling, although liver damage was reduced. These results imply that hepatic IL-6 signaling may increase liver inflammation but also protects against the development of hepatic steatosis [42]. A possible causative relationship between I148M-mediated sensitivity to NAFLD and increased IL-6/STAT3 activity [43]. The alterations in the blood’s concentration of soluble IL-6 receptors were linked to the advancement of NAFLD. Additionally, a strong association between alterations in soluble IL-6 receptor levels and hepatocyte and peripheral blood leukocyte death was shown [44]. The presence of hyperglycemia and hyperlipidemia may directly impact IL-6 trans-signaling, potentially contributing to the increased severity of NAFLD in individuals with concurrent diabetes [45]. Animal models indicated that increased hepatic expression of IL-6 was linked to NAFLD, the hypothesis was that IL-6 can serve as a predictor of NAFLD progression [48]. IL-6 showed an elevation specifically in NASH cases [50].
IL-7	IL-7 decreased with portal inflammation and fibrosis severity [30].
IL-8	Patients with lobular inflammation and stage 3–4 fibrosis had higher levels of IL-8 [30]. Compared to people with a normal liver, morbid obesity with NASH was associated with considerably higher levels of IL-8 [55]. While the A/A genotype in the IL-8 gene was linked to the advancement of the illness, polymorphisms in the IL-6 and IL-8 genes do not appear to have a role in the pathophysiology of NASH or liver fibrosis [56]. IL-8 plays a crucial role in systemic inflammation progression and, more importantly, contributes significantly to the escalating severity of liver disease in NAFLD [57]. In HFD-fed mice, hepatic overexpression of human IL8 stimulates neutrophil infiltration and accelerates the transition from fatty liver to NASH [58]. IL-8 was shown to be a robust predictor of enhanced fibrotic liver damage compared to recognized markers of hepatic fibrosis [60]. IL-8 levels were higher in patients with liver fibrosis among those with obesity and NAFLD having bariatric surgery [59].
IL-9	In rats with NAFLD, taurine may prevent or treat liver and ileal mucosa damage by downregulating the production of TGF-β and IL-9. These two factors may be involved in the development of NAFLD [62].
IL-10	No difference between IL-10 between NAFLD patients and controls was shown [33]. IL-10 is an independent factor associated with NAFLD [51]. IL-10 is a major regulatory factor in the pathophysiology of NAFLD, as well as in inflammatory illnesses, malignancies, and autoimmune disorders [64]. IL-10 and IL-1β may be taken into consideration as biomarkers of the severity of NAFLD [66].

## Data Availability

Data are contained within the article.
